# Bioprinted 3D Bionic Scaffolds with Pancreatic Islets as a New Therapy for Type 1 Diabetes—Analysis of the Results of Preclinical Studies on a Mouse Model

**DOI:** 10.3390/jfb14070371

**Published:** 2023-07-14

**Authors:** Marta Klak, Michał Wszoła, Andrzej Berman, Anna Filip, Anna Kosowska, Joanna Olkowska-Truchanowicz, Michał Rachalewski, Grzegorz Tymicki, Tomasz Bryniarski, Marta Kołodziejska, Tomasz Dobrzański, Dominika Ujazdowska, Jarosław Wejman, Izabela Uhrynowska-Tyszkiewicz, Artur Kamiński

**Affiliations:** 1 Foundation of Research and Science Development, 01-793 Warsaw, Poland; marta.klak@fundacjabirn.pl (M.K.);; 2 Polbionica Sp. z o.o., 01-793 Warsaw, Poland; 3 Chair and Department of Histology and Embryology, Medical University of Warsaw, 02-004 Warsaw, Poland; 4 Department of Transplantology and Central Tissue Bank, Medical University of Warsaw, 02-004 Warsaw, Poland; 5 Center for Pathomorphological Diagnostics Sp. z o.o., 01-496 Warsaw, Poland

**Keywords:** diabetes, pancreas islets, 3D bioprinting, glucose, xenotransplantation, bionic scaffold

## Abstract

Recently, tissue engineering, including 3D bioprinting of the pancreas, has acquired clinical significance and has become an outstanding potential method of customized treatment for type 1 diabetes mellitus. The study aimed to evaluate the function of 3D-bioprinted pancreatic petals with pancreatic islets in the murine model. A total of 60 NOD-SCID (Nonobese diabetic/severe combined immunodeficiency) mice were used in the study and divided into three groups: control group; IsletTx (porcine islets transplanted under the renal capsule); and 3D bioprint (3D-bioprinted pancreatic petals with islets transplanted under the skin, on dorsal muscles). Glucose, C-peptide concentrations, and histological analyses were performed. In the obtained results, significantly lower mean fasting glucose levels (mg/dL) were observed both in a 3D-bioprint group and in a group with islets transplanted under the renal capsule when compared with untreated animals. Differences were observed in all control points: 7th, 14th, and 28th days post-transplantation (129, 119, 118 vs. 140, 139, 140; *p* < 0.001). Glucose levels were lower on the 14th and 28th days in a group with bioprinted petals compared to the group with islets transplanted under the renal capsule. Immunohistochemical staining indicated the presence of secreted insulin-living pancreatic islets and neovascularization within 3D-bioprinted pancreatic petals after transplantation. In conclusion, bioprinted bionic petals significantly lowered plasma glucose concentration in studied model species.

## 1. Introduction

Recently, tissue engineering, including 3D bioprinting of the pancreas, has acquired clinical significance and has become an outstanding potential method of customized treatment for type 1 diabetes mellitus [1]. The use of donor-derived islets in 3D-bioprinted constructs can be exploited faster in clinical trials than stem cell-derived islets or knocked-out pig-derived islets. However, before the tissue engineering-based approach may be effectively used, emerging problems such as the islets of Langerhans isolation, transplantation method, and maintaining conditions in the new environment need to be improved [2,3]. The harsh isolation conditions put the islets under numerous stress factors, including exposure to the cytotoxicity of the matrix degradation enzymes and cell–cell signaling alteration [4,5] but also devascularization, ischemia, or oxidative stress [6]. 

The first stage of the islet isolation process aims at ECM (extracellular matrix) degradation, and disruption of the cell–ECM binding seems to destroy what is crucial in the preservation of islet function and identity. The maintenance of the proper microenvironment of islets after the isolation process is the next limiting step that influences process effectiveness and islet function. The extracellular matrix of the pancreas is mainly composed of collagen type I, III, IV, V, and VI, elastin, as well as laminin and fibronectin [3]. Tissue-specific scaffolds (constructs), which are composed of biopolymers, are often used to mimic the native pancreatic environment [7,8]. In particular, the components of hydrogel biopolymers, such as collagen, gelatin, and hyaluronic acid, create the pancreatic-like microenvironment favoring islets culture but also providing adhesion and stimulating cell proliferation, differentiation, and viability [9]. 

The decellularization process of the target tissue delivers the ECM of a suitable quality that maintains all biochemical and functional properties of pancreatic-specific scaffolds [10]. Nevertheless, the decellularization procedure of a whole organ is the subject of ongoing studies. The preservation of the structural and biomechanical properties of the native ECM and simultaneously removing the cellular fraction containing DNA and surface antigens is essential for the process of cell colonization and implantation [11].

The authors developed an innovative approach involving the production of biomaterials and scaffolds that significantly improve the functionality of pancreatic islets isolated from the native organ. As part of extensive research, the authors solved serious problems related to the process of optimizing the printing parameters of bionic models and the composition of bioinks used for bioprinting with living cells or microorgans (such as pancreatic islets). As part of the research, the optimal composition of bioink was demonstrated, which was extensively analyzed in terms of physico–chemical and rheological properties as well as biological properties. As part of the biological studies, the 3D functionality of bionic scaffolds was assessed in vitro, as well as the cytotoxicity and vascularization potential of scaffolds in an in vivo model. The results of the study showed that: the bioink produced by the authors allows for bioprinting of stable constructs with biological material, and the optimized composition of the bioink significantly improves the functionality of pancreatic islets. This is evidenced by the fact that the full functionality of pancreatic islets in vitro was maintained for 7 days. In addition, the bioink does not show any cytotoxicity for the cells suspended in it and the organism into which it is implanted. And, most importantly, from the point of view of practical tissue engineering, the process of vascularization in the scaffold was observed just 8 weeks after implantation in the mouse. Vascularization of bionic tissue systems is important due to the possibility of supplying nutrients and oxygen exchange and metabolites in constructs thicker than 200 µm. Without such properties, the practical implementation of bioprinted tissues and organs in medical practice will not be possible [12].

Our study aimed to evaluate the functionality of designed bionic, 3D-bioprinted pancreatic scaffolds with islets of Langerhans in the xenotransplantation model. Hereby, for the first time, we present the results of in vivo studies on the murine model of bioprinted constructs with embedded pancreatic islets.

## 2. Materials and Methods

### 2.1. Research Model and Transplantation

**Figure 1:**
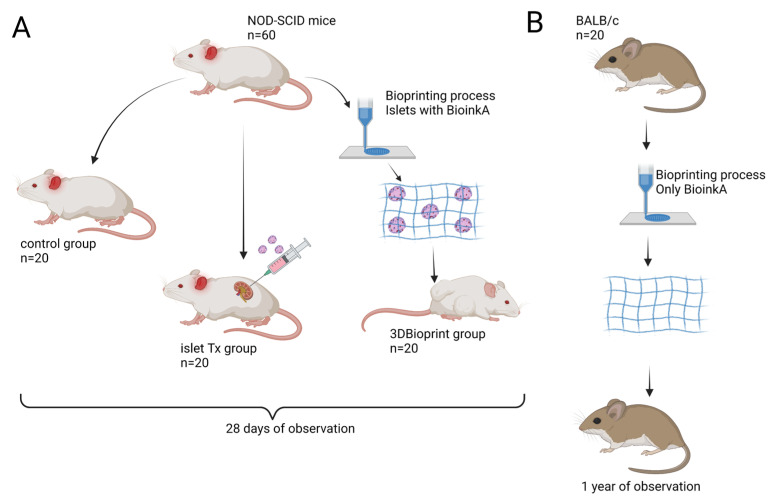
Schematic presentation of the conducted experiment. The conducted experiment was aimed at analyzing two main goals: (**A**) functional assessment of bioprinted pancreatic islets in an in vivo model; (**B**) analysis of the occurrence of a planned state in response to the implanted biomaterial (without pancreatic islets). Created with www.biorender.com (accessed on 11 July 2023).

The research was carried out on 60 NOD-SCID mice and 20 BALB/c mice that were 8 weeks old (Figure 1). The authors used both of these strains in their research because they have the genetic background of each other. In this way, the authors maintained the consistency of the individual stages of the research. However, the authors point out that the research on the BALB/c model and the NOD-SCID model had different purposes. In NOD-SCID mice, the goal was to demonstrate the functionality of the implanted constructs. In BALB/c mice, the occurrence of infiltration of such cells as lymphocytes, macrophages, and monocytes was assessed. 

The research was conducted following the ARRIVE guidelines [13] and approved by the Ethics Committee No. 1 of the Faculty of Biology, University of Warsaw, consent no. 461/2017. The number of animals used for the experiments was calculated in such a way as to ensure reliable results and to be able to perform statistical analyses. It was determined using statistical methods. To perform statistical analyses, the following criteria were adopted: test power = 0.9 and statistical significance at the level = 0.05.

The NOD-SCID mice were divided into 3 groups: (1) control; (2) IsletTx, in which porcine pancreatic islets were transplanted under the renal capsule; (3) 3D bioprint, in which transplantation of bioprinted petals was performed. Induction of diabetes within transplanted groups was not performed. The control group consisted of healthy mice with a properly functioning native pancreas. The petals were transplanted into the dorsal part of the muscles under the skin. Each transplant in both groups consisted of 3000 iEq (islet equivalents). The body mass of each animal was analyzed before the experiment and after one month.

Additionally, the study was enriched with tests on BALB/c mice, which are widely used in research on implantation and biocompatibility of biomaterials [14,15,16]. 

### 2.2. Bioink Preparation

To prepare a dECM (decellularized extracellular matrix)-based bioink, a process of decellularization of the pancreas was performed with Triton X-100 detergent according to the protocol described by Klak et al., 2021 [11]. dECM pancreatic powder obtained in this way was used for further steps. It was dissolved in 0.01 M hydrochloric acid (Merck Millipore, Darmstadt, Germany) with pepsin (European Pharmacopoeia Reference Standard, Strasbourg, France ) on a magnetic stirrer for 72 h at room temperature. The activity of pepsin was stopped by adding 0.1 M sodium hydroxide (Sigma-Aldrich, Saint Louis, MI, USA) and PBS for ionic strength balance. Gelatin methacrylate (GelMA) and methacrylic hyaluronic acid (HAMA) provided by Polbionica Sp. z o.o. (Warsaw, Poland) dissolved in PBS with phenyl-2,4,6-trimethyl-benzoyl phosphinate (LAP) photoinitiator (Polbionica Sp. z o.o., Warsaw, Poland) and used as a crosslinking agent capable of crosslinking in UV-vis light (405 nm). A blend of dECM, GelMa, HAMA, and LAP was considered as a bioink for extrusion bioprinting. 

### 2.3. Source of Porcine Pancreatic Islets 

Pancreas were derived from adult, 12-month-old, 55–70 kg male pigs (domestic pigs). The research material was obtained from a local slaughterhouse. To ensure the homogeneity of the biological material for implantation, scaffolds or islets (implanted under the renal capsule) were made from one isolation process for 20 animals at a time (10 for the renal capsule and 10 for bioprinting of the scaffolds). Due to the number of animals used for the study, the isolation procedure was performed twice. Organs were dissected, preserved in cold UW solution (University of Wisconsin cold storage solution), and immediately transported to the laboratory. Islets were isolated from the native organ using collagenase NB8 (Nordmark Pharma, GmbH, Deutschland, Germany) and a Ricordi chamber. The next step was their purification with COBE (cell separator; COBE 2991) and 1108 and 1069 gradients. In one process, 300–500,000 islets were isolated and left to culture overnight before being used for future experiments. The viability of the islets was assessed before implantation. For this purpose, FDA/Pi staining (Fluorescein Diacetate Sigma-Aldrich, Saint Louis, MI, USA and Propidium Iodide Cayman Chemical, Ann Arbor, MI, USA) and a secreted insulin-specific ELISA (Mercodia, Uppsala, Sweden) test were performed.

### 2.4. Three-Dimensional Bioprinting of Bionic Petals

**Figure 2:**
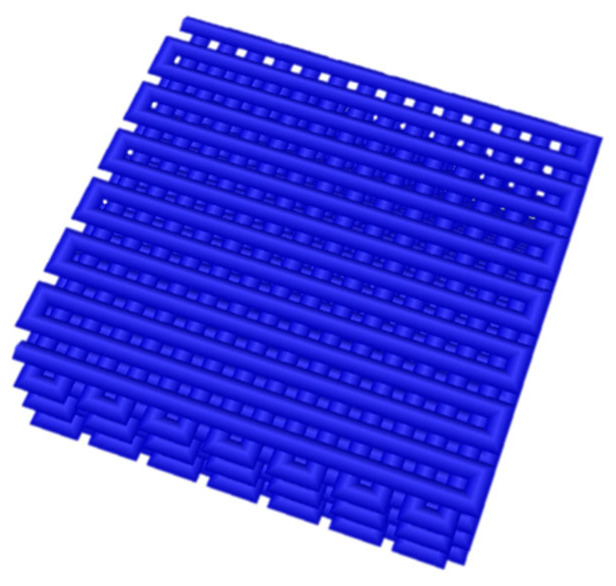
CAD illustration of bionic scaffolds transplanted to an animal model. The model consists of successively superimposed layers, with the layers printed at an angle of 90° to each other.

The bionic petals were bioprinted with the extrusion technique [12,17,18]. The bioink that was used to print the bionic scaffolds consisted of natural ingredients (dECM) [11], methacrylic gelatin (GelMa; Polbionica Sp. z o.o., Warsaw, Poland), methacrylic hyaluronic acid (HAMA; Polbionica Sp. z o.o., Warsaw, Poland), and lithium phenyl-2,4,6-trimethyl-benzoyl phosphinate photoinitiator (LAP; Polbionica Sp. z o.o., Warsaw, Poland) as a crosslinking component [19]. Crosslinking with visible light (405 nm) was applied while bioprinting [20]. Directly before the bioprinting process, isolated pancreatic islets (in the amount of 3000 iEq per 1 scaffold) were mixed with bioink. Bioink with islets was transferred to the cartridge and the bioprinting process was ordered according to the developed g-code (Figure 2).

The CELLINK^®^ BIOX 3D bioprinter (CELLINK, Gothenburg, Sweden) was used for bioprinting. The petals were 1 cm^2^ in size and consisted of 7 layers, each 600 µm thick (Figure 1). The temperature of the print bed (20 °C) and the printhead (18 °C) were controlled continuously. The pressure during the bioprinting process did not exceed 30 kPa to maintain the highest possible viability in the pancreatic islets. The printing speed was 20 mm/s. The nozzle diameter was constant for all constructs at 609 µm. The size of the nozzle was determined by biological material, as the pancreatic islets are up to 500 µm in size. In addition, when using such a nozzle, the highest viability and functionality of the pancreatic islets were demonstrated. The results of these studies were published by Klak et al. [17].

### 2.5. Blood Analytical Research

#### 2.5.1. Glucose Measurement

Glucose measurements were performed with a glucometer using blood collected after the procedure of tail incision. All animals were deprived of solid food two hours before blood sampling. Glucose was analyzed before the experiment and after surgery on 7, 14, and 28 days. A Wellion CALLA Light glucometer (Symphar Sp. z o.o., Wasaw, Poland) was used to measure glucose concentration. 

#### 2.5.2. Peptide-C Measurement 

Mouse C-peptide was analyzed. To determine the concentration of C-peptide in serum, measurements were taken before the experiment and 7, 14, and 28 days after the transplantation of islets or bionic scaffolds. The mouse C-peptide ELISA (ALPCO; 80-CPTMS-E01) was used for this purpose. The amount of serum collected from animals was sufficient and did not require dilution. 

### 2.6. Microscopic Analysis

Samples were collected and fixed for 48 h in 4% (*v*/*v*) paraformaldehyde. Following paraffin embedding, samples were cut into a 5 μm thick section. Then, the sections were deparaffinized in xylene (Chemsolve WITKO, Lodz, Poland) for 5 min and rehydrated by immersing the sections in several ethanol dilutions (95%, 85%, 70%, and 50%) for 2 min each. Then, sections were rinsed in distilled water, and appropriate staining was performed, including standard hematoxylin-eosin (H&E, Merck, Darmstadt, Germany) stain for histologic examination. 

The imaging was performed under an Olympus microscope IX83 (Olympus Corporation, Tokyo, Japan) with magnifications 20- and 40-fold using CellSene Dimension software (Version 1.17, Olympus Corporation, Tokyo, Japan). Micrographs were taken using bright-field or blue fluorescent for visualization of nuclei.

#### 2.6.1. Immunohistochemistry and Imaging of Bionic Scaffolds and Kidney

The heat-induced epitope retrieval using microwave in citrate buffer (10 mM citric acid, 0.05% Tween 20, pH = 6.0, Merck, Darmstadt, Germany) was performed. When needed, tissue sections were incubated in 3% H_2_O_2_ in TBST (Tris-buffered saline (TBS) solution with the detergent Tween 20, ABO Gdanska, Poland) buffer for 30 min. After blocking in 10% normal horse serum for 2 h RT (room temperature), the tissue section was incubated with primary antibodies: insulin (1:100 dilution, sc-8033; Santa Cruz Biotechnology, Dallas, TX, USA), glucagon (1:100 dilution, sc-514592; Santa Cruz Biotechnology, Dallas, TX, USA), CD45 (Novus Biologicals, Centennial, CO, USA, NB100-77417, 1:500), or MOMA (Novus Biologicals, Centennial, CO, USA; NB100-64946, 1:500) overnight at 4 °C. The tissue section used as the negative control was incubated with 10% normal horse serum overnight at 4 °C. Then, after washing, the sample sections were covered by AP- or HRP-conjugated secondary mouse antibody (Vector-ImmPRESS Reagent Kit, MP-5402, or Vector-ImmPRES Duet, MP-7724; Vector Laboratories, Newark, CA, USA) for 2 h RT. Following the three washing, the AP/DAB substrate (Vector-ImmPRES Duet, MP-7724, Vector Laboratories, Newark, CA, USA) was added to the tissue slides. When staining was well developed, slides were washed and then mounted.

#### 2.6.2. DAPI Staining of Nuclei

The autofluorescence quenching kit (Vector-TrueVIEW, SP-8400, Vector Laboratories, Newark, CA, USA), according to the manufacturer’s protocol, was used to reduce the very high autofluorescence of scaffolds derived from the proteins of ECM. Then, the slides were washed in TBST buffer and mounted with a mounting medium with Antifade Mounting Medium with DAPI to visualize the nuclei (Vector-Vectashield, H-1200, Vector Laboratories, Newark, CA, USA).

### 2.7. Statistical Analysis

The significant difference from the respective controls for each experimental test condition was assessed by one-way analysis of variance (ANOVA) and the Dunnett test. The difference is significant if the *p*-value is less than 0.05.

## 3. Results

### 3.1. Body Weight Measurements 

Body weight analysis showed no difference between the experimental groups. There were also no significant changes in the weight of animals within a given research group. In the control group, the average body weight on the day of the experiment was 23.7 g, while on the 28th day of the experiment, it was 23.8 g. In the IsletTx group, the weight gain was, on average, 0.13 g, and in the 3D-bioprint group, 0.11 g.

### 3.2. Mean Fasting Glucose Analysis

**Figure 3:**
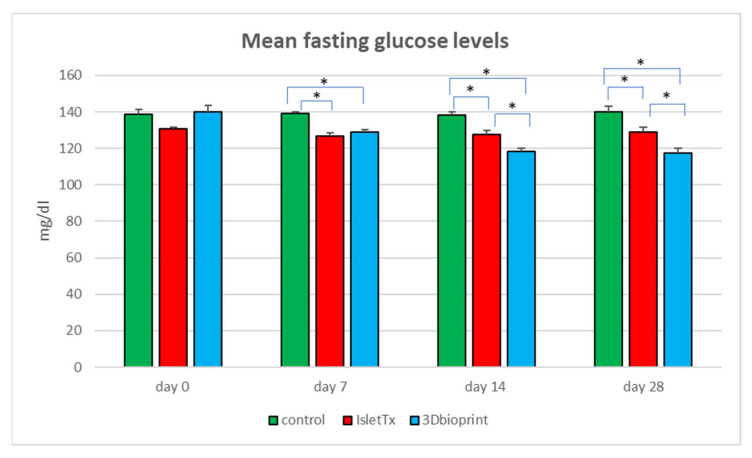
Plasma glucose levels. Mice fasted overnight, and then the blood samples were taken, and glucose level was measured at starting points and then in four checkpoints: after 7, 14, and 28 days. The bars represent the mean of mice from the control group (green), IsletTx-injected group (red), and mice group with implanted 3D bioprint (blue). The error bars represent ± SD, * < 0.05.

Studies conducted on a mouse model showed significant differences between the mean glucose levels in the studied group of animals (Figure 3 and Table 1). The main goal was to investigate the effectiveness of bionic scaffolds in preserving normoglycemia in studied animals. Furthermore, the functionality of the printed bionic scaffolds was compared to the control group of studied animals and to a group where transplantation of islets under the renal capsule was performed. The group with transplanted petals (3D bioprint) showed significantly lower mean glucose levels on the seventh day after the procedure. The difference between the groups was over 7%. After 14 days of analysis, the 3D-bioprint group still showed lower glucose values. However, at this point, significance was demonstrated for the control group, where the difference in the obtained results was almost 15% (*p* < 0.0001), and in the IsletTx group (*p* < 0.0001); the difference was 7.4%. These parameters were kept until the end of the experiment. On day 28, the lowest glucose level was found in the group with bionic scaffolds (control vs. 3D bioprint, *p* < 0.0001 and IsletTx vs. 3D bioprint, *p* = 0.0002), where the differences ranged from 9% for the IsletTx group to almost 17% for the control group. The islets transplanted under the renal capsule also showed lower glucose levels compared to the control group (*p* = 0.0003).

Statistical analysis also showed significant differences within the 3D-bioprint group. Already on day 7 after implantation, significantly lower glucose parameters were demonstrated (day 0 vs. day 7, *p* < 0.0001). Detailed data are presented in Table 1. In the control group and IsletTx group, the changes were not significantly noticeable.

### 3.3. Mean Fasting Mice C-Peptide Results

**Figure 4:**
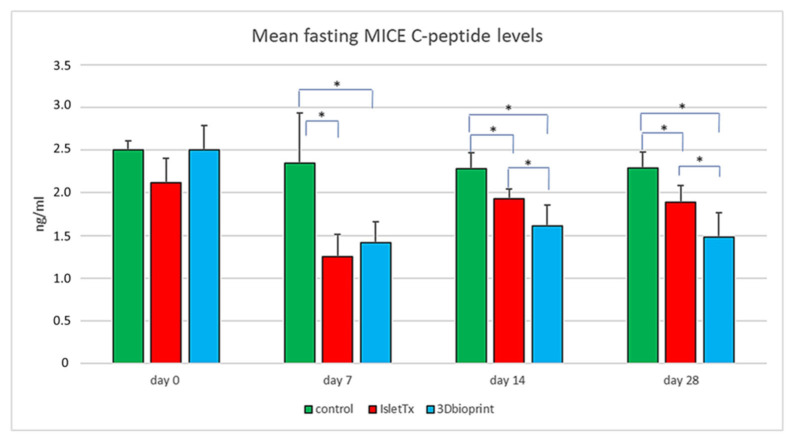
Plasma C-peptide levels. Mice fasted overnight, and then the blood samples were taken, and the C-peptide level was measured at starting points and then at four checkpoints: after 7, 14, and 28 days. The bars represent the mean of mice from the untreated-control group (green), IsletTx-injected group (red), and mice group with implanted 3D bioprint (blue). The error bars represent ± SD, * < 0.05.

Analysis of murine C-peptide concentration showed a significant decrease in serum levels in the groups subjected to transplantation from day 7 after surgery (Figure 4 and Table 2). Both in the group of animals with islets implanted in the bionic scaffolds and under the renal capsule, a lower level of C-peptide was observed already at the first measurement point (IsletTx vs. control; *p* < 0.0001 and 3D bioprint vs. control; *p* = 0.0002). On day 14 after surgery, it was shown that IsletTx vs. control, *p* = 0.293, and 3D bioprint vs. control, *p* = 0.0008. Additionally, there was a significant difference between the study groups (IsletTx vs. 3D bioprint; *p* = 0.0422). This tendency continued until the end of the experiment. On day 28, the following relationships between the groups were noted: IsletTx vs. control, *p* = 0.364; 3D bioprint vs. control, *p* = 0.0007; and IsletTx vs. 3D bioprint, *p* = 0.0305.

Similarly to the results obtained for glucose measurements, in the case of the C-peptide, significant differences were also found within the given study groups (Table 2). Changes were not shown only in the control group, where the concentration of the C-peptide remained constant throughout the entire time. 

In summary, the results of the study show the functionality of xenotransplanted 3D bionic scaffolds with pancreatic islets. The effect of the functionality of transplanted pancreatic islets is observed due to the lower level of mouse C-peptide in both the IsletTX and 3D bioprint groups. 

### 3.4. Analysis of 3D-Bioprinted Scaffolds 

**Figure 5:**
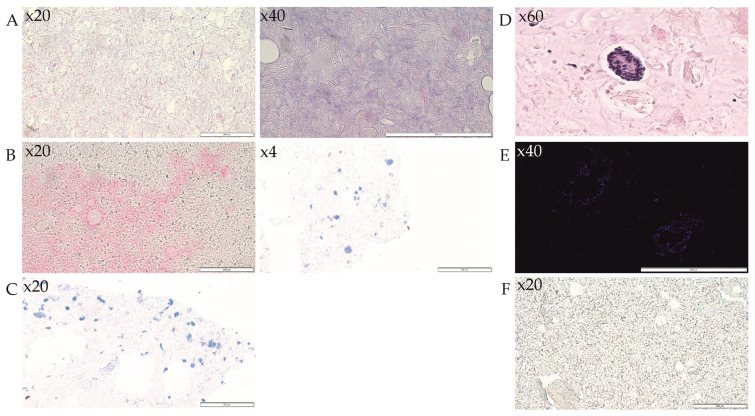
Histological examination of a cross-section of in vitro scaffolds. The representative pictures of (**A**,**D**) hematoxylin-eosin (morphology), (**B**) insulin, and (**C**) glucagon staining are shown. On the micrograph (**D**), hematoxylin-eosin and (**E**) DAPI staining of islet immersed in the scaffold is visualized. The micrograph (**F**) is presented negative control for the immunohistochemistry staining. The micrographs were taken using Olympus microscopy at a magnification of 4-, 20-, 40-, and 60-fold. The scale bars represent 200 μm.

Insulin revealing was performed to confirm the islets’ secretory function (Figure 5). The general morphology of the scaffolds was studied with H&E staining (Figure 5A,D). The insulin (Figure 5B) and glucagon (Figure 5C) revealing were performed to confirm the islets’ secretory function. The insulin staining has confirmed that islets immersed in the bioink express insulin, which is then dispersed in the surrounding ECM. Insulin concentration is present over the sections surface of the scaffolds (Figure 5B). However, glucagon secretion occurs in a small area, probably due to the lowest level of expression. 

Further, the islet morphology and the cell nuclei were confirmed with H&E (Figure 5D) and DAPI (Figure 5E) staining, confirming that the immersed islets save the function and identity of alpha and beta cells.

### 3.5. Analysis of Islets Transplanted under the Renal Capsule

**Figure 6:**
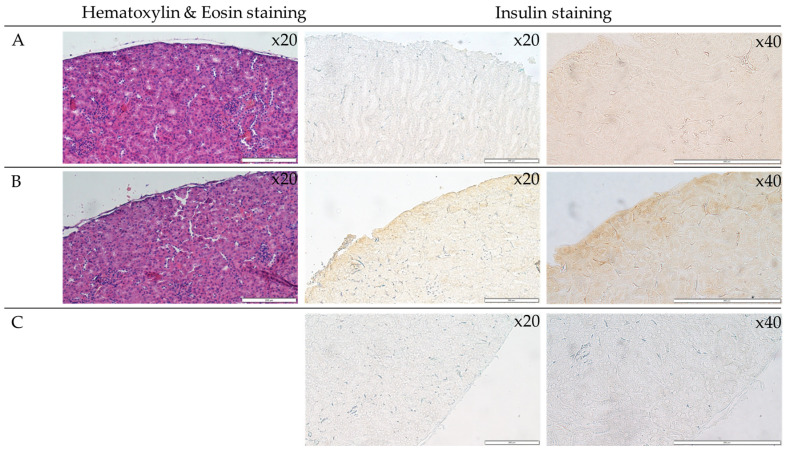
The kidney morphology. The representative pictures of hematoxylin-eosin and insulin staining are shown. The representative micrographs of the tissue staining are presented: the control kidney (**A**), the operated kidney (**B**), and the negative control for the immunohistochemistry (**C**). The micrographs were taken using Olympus microscopy at a magnification of 20- and 40-fold. The scale bars represent 200 μm.

The transverse sections of the kidneys were stained with hematoxylin-eosin dye. The H&E staining of the kidney sections has visualized the tissue morphology, which was not affected after surgical intervention (Figure 6). However, the insulin detection across the tissue section revealed insulin expression in the post-surgery kidney (Figure 6B). These results suggest the presence of insulin secreted by the islets in the examined tissue (brown color).

### 3.6. Immunohistochemical Analysis of Monocytes and Macrophages (MOMA) within the Bionic Scaffolds and Postoperative Tissues from Mice

**Figure 7:**
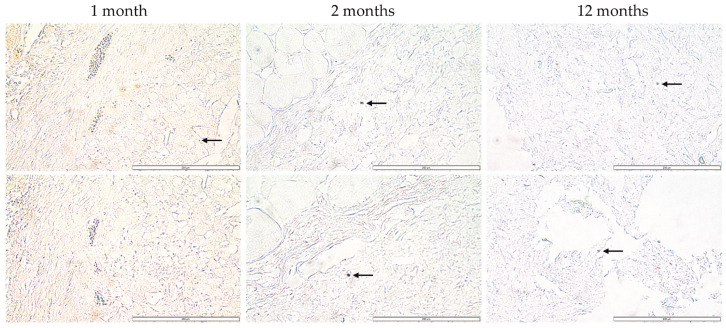
Immunohistochemical analysis of postoperative tissue. Cells infiltrating the postoperative bionic construct were stained brown (DAB). In the photos, stained cells are indicated by an arrow. Imaging is from the Olympus microscope at ×40 magnification. The scale bars represent 200 μm.

Imaging was performed on the scaffolds removed after 4 weeks, 8 weeks, and 12 months from implantation (consisting of only bioink A, without islets). The analysis showed the presence of only single monocytes/macrophages, which is evidence of an inflammatory lesion in the examined postoperative tissues (Figure 7). This result was obtained at all three time points, i.e., after 4 and 8 weeks as well as one year after implantation. Microscopic observation showed that the stained cells do not exist in colonies but only as single cells, which suggests that they patrol the examined tissues but do not accumulate in them. The presented results suggest that the bionic constructs implanted subcutaneously in mice did not induce increased infiltration and accumulation of monocytes/macrophages and did not induce an inflammatory state within the operated site. 

### 3.7. Immunohistochemical Analysis of Lymphocytes (CD45) within the Bionic Scaffolds and Postoperative Tissues from Mice

**Figure 8:**
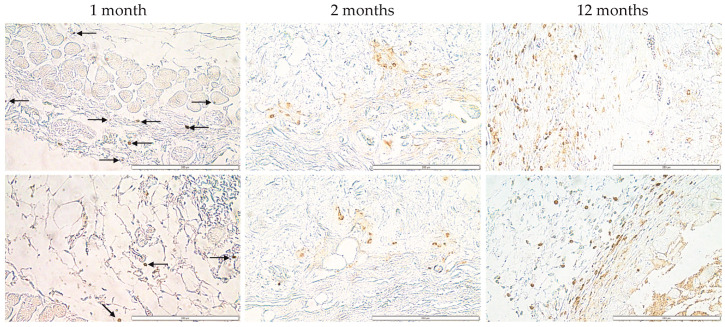
Immunohistochemical analysis of postoperative tissue from mice. Cells infiltrating the postoperative flap were stained brown (DAB). Imaging is from the Olympus microscope at ×40 magnification. The scale bars represent 200 μm.

The imaging showed only single lymphocytes (CD45-positive cells), which proves the absence of an inflammatory focus within the tested preparation. On the other hand, the result obtained 12 months after implantation was characterized by greater infiltration of CD45-positive cells. Lymphocyte infiltrates were mainly located in the vicinity of the bionic scaffolds (Figure 8). However, only a few stained cells were found within the bionic construct itself.

## 4. Discussion

Before starting the functional tests on animals, the authors tested the bioink composition they propose, both under in vitro and in vivo conditions, for the cytotoxicity of the biomaterial itself. In vivo studies have also been performed in a mouse model [12]. Mice are a popular research model used in the first step of the preclinical research phase. However, in the context of type 1 diabetes, this model is not perfect, especially because of the difficulty of developing a stable model of diabetes [21]. The issue of the significant instability of the type 1 diabetes model induced by STZ (streptozotocin) injection in mice was raised by the team of Wszoła et al. [22]. In addition, mice injected with STZ showed glomerulonephritis, and liver tissue showed large necrotic areas. Based on the analyses carried out by the team of Wszoła et al. and the analysis of the mortality rate of animals, it was shown that the induction of type 1 diabetes with STZ in BALB/c mice is not an appropriate method to induce such a research model [22], which has also been confirmed by other scientific teams. Graham et al. [23] showed that the induction of diabetes in mice by STZ also caused severe and common side effects. On this basis, it was decided that the study evaluating the functionality of 3D-bioprinted scaffolds with pancreatic islets did not involve inducing diabetes with STZ. Importantly, independent studies have shown that the use of porcine pancreatic islets for testing does not allow the evaluation of porcine C-peptide to assess the functionality of the implanted pancreatic islets [24]. This confirms the validity of the author’s approach to the analysis of transplanted constructs. Measuring C-peptide concentration in peripheral blood is considered a principal and primary tool for the evaluation of insulin production by transplanted islets or grafts [25,26,27]. Nevertheless, difficulties related to providing reliable measurements might be confused with non-functional grafts [28]. Thus, it was noted that the concentration of C-peptide in peripheral blood might be very low and often undetectable, which might lead to a false negative assessment of transplant functionality [29,30]. Such an observation was put forth by Nishimura et al. [28], where encapsulated porcine islets were intraperitoneally transplanted for 3–6 months into STZ-induced diabetic male nude mice (BALB/cA Jcl-nu/nu). The authors proved that transplanted islets, while retrieved from mice, were functional and viable; however, C-peptide concentrations in blood were not coherent with this observation. Transplanted islets secrete insulin directly in a place of transplantation where it might be used locally, whereas a very limited amount of it enters the stream of peripheral blood [28]. Gazda et al. [31] have shown that porcine C-peptide was detectable in peritoneal fluid, where transplantation was performed, but in serum, C-peptide was not detected. It remains unclear what occurs to xenogenic C-peptides as they move from the peritoneal cavity to peripheral blood. Nevertheless, Nishimura et al. conclude that even at low concentrations of porcine C-peptide, the glucose concentrations in peripheral blood are reduced, probably because insulin concentration in the peritoneal cavity is sufficient. The team of Cosimi et al. [32] showed significant differences in the functioning of the islets as well as their sensitivity to glucose concentration. They showed that the release of insulin was higher in the case of material from rodents than in pigs. The results of studies presented by Cosimi et al. showed that insulin release at low (3.3 mmol/L) glucose concentration is higher in rats than in large mammals. In addition, differences in the sensitivity of the islets to fluctuating glucose levels have been demonstrated. Rodent islets turned out to be the most sensitive to rising glucose levels. Porcine islets showed maximum insulin secretion at a mean glucose concentration (14 mmol/L) without the additional effect of higher concentrations. In contrast, bovine islets only responded to 25 mmol/L glucose. These studies show that islet interspecies transplantation requires additional analysis and that it is not possible to rely on just one parameter (glucose level or C-peptide). The results must be analyzed correlatively. Those data explain why in clinical practice, transplanted xenogenic islets are associated with limited C-peptide concentration in peripheral blood [33]. Doubling the weight of pancreatic islets in mice is supposed to lower glucose levels as well as reduce the function of the mice’s pancreatic islets, as porcine islets should take control of blood sugar metabolism; this effect is seen in our study in both groups with transplanted porcine islets, lower average glucose level, and mice C-peptide in both groups, which clearly confirms the hypothesis and shows the suitable function of the transplanted bionic scaffolds with 3D-printed porcine islets. Surprisingly, Cui et al. [34] succeeded in the detection of porcine C-peptide in murine blood, which is a contradicting result presented in this study and the study of Nishimura. However, the key difference in these studies concerns the mouse model and the number of implanted islet counterparts and the type of islets, as Cui’s team [34] used adult islets for their research, and Nishimura’s team [28] used neonatal porcine islets. Genetically modified pigs are proposed to be used as a source of islets for xenotransplantation to achieve the C-peptide amino acid homogeneity with the recipient species [35]. Hence, xenograft function may be underestimated when its functionality is assessed on limited factors, especially C-peptide concentration [24,28]. 

Furthermore, metabolism pathways and kinetics of xenogeneic C-peptide are crucial to understanding the dynamics of proinsulin [36,37]. It was suggested by Wennberg et al. [24] that the production of antibodies against xenogeneic C-peptide might be difficult to induce due to the differences in molecular weight of autologous and xenogenic C-peptides. The explanation for this phenomenon might be related to the fact that xenoreactive antibodies are directed mainly toward carbohydrate antigens [38,39], whereas the studied C-peptide appeared to be glycosylated. It is also proved that improved glycemic control after islet transplantation is not associated with C-peptide concentration. Moreover, it was noticed that the concentration of porcine C-peptide after intraperitoneal injection into rodents decreases rapidly while measuring it from peripheral blood. Surprisingly, C-peptide concentration is 1/40 of that when C-peptide is injected intravenously [40]. This finding suggests that when C-peptide (and insulin in about equal amounts) is released from transplanted islets, only 1/40 of its concentration is expected to be detected. The terminal elimination half-life of porcine C-peptide, just like most peptides, is very short [40,41]. Interestingly, it was shown that the value for porcine C-peptide, while injected intravenously, varies from around 4 to 9 min. This means that research on the low concentration level of C-peptide is prone to misjudgment. Moreover, C-peptide slowly diffuses into the venous circulation, but as soon as it gets there, its quick elimination occurs. This might be the probable explanation for significant differences in C-peptide concentrations between peripheral blood and the area where transplantation occurred, e.g., ascetic fluids. This discovery seems to shade a new light on judgment about islets graft function failure. Thus, problems with detection of xenogeneic C-peptide should not be the basis of transplant validation, as soon as normoglycemia is present. Yet, the possible metabolic pathway of xenogeneic C-peptide is speculative; however, it might lay on the root of recipient species’ enzymatic activity. 

However, despite these reports, the authors decided to conduct research using porcine pancreatic islets and demonstrate the functionality of bionic pancreatic constructs. There is a difference in the anatomy of pancreatic islets between species: mice and pigs islets differ in their morphology and distribution of specific cells within an islet [42,43]. However, despite these differences, pig islets are the most anatomically and functionally similar to human islets. Therefore, the authors decided to use them in this study [44]. Moreover, in light of the available studies, it was shown that porcine islets are as functional as mouse islets, so they can be used in studies in a mouse model. Therefore, by providing each mouse with an additional 3000 pancreatic islets, it was expected that the islets in a well-functioning pancreas would be less active. Therefore, the analysis of the C-peptide and glucose levels in fasting mice allowed the determination of the functionality of the bionic scaffolds. Their functionality was demonstrated by decreasing the concentration of mouse C-peptide and lowering glucose levels. The results of glucose and C-peptide measurements confirmed this assumption and thus became another milestone in research into new therapies in T1D (type 1 diabetes) [44,45,46]. 

There is no doubt that pancreas or pancreatic islet transplantation is currently the only method of treating people with type 1 diabetes who suffer from severe complications. Islets isolation and transplantation have shown great progress in development in recent years [47]. However, despite intensive progress in the field of transplantation, transplantation of pancreatic islets or the entire pancreas is still not an ideal solution. Therefore, transplantology is more and more willing to cooperate with other scientific fields, e.g., tissue engineering using 3D-bioprinting technology from natural polymers [47]. It has been shown that the destruction of the extracellular matrix (ECM) and the dense vascular network during islet isolation is one of the main causes of their decreased functionality [48]. Therefore, the restoration of the native pancreatic microenvironment and its quick revascularization should be the factors that will positively affect their condition.

Hereinafter, the adaptation of the islets isolation process and ECM composition resulted in high viability and suitable maintenance of the secretory function of islets. Islets not only did not undergo dissociation after the isolation and bioprinting process but remained functional (Figure 6) and present islets-like morphology (Figure 6). On the other hand, the secretion of insulin confirms that the ECM-based bioink A provides the optimal condition of islet culture in vitro and in vivo after scaffold implementation.

Despite the fast development of tissue bioprinting technology, very little is known about the viability of islets in post-surgery scaffolds. Moreover, the bioink formulation is still in research. Besides ECM-based bioinks, an alginate-based composite material (alginate/methylcellulose) was tested in the in vitro studies in terms of bioprinting of rat pancreatic islets. Duin et al. [47] have shown that after the successful incorporation of islets into alginate/methylcellulose scaffolds, the viability was not changed until 7 days of culture. However, the apoptotic nuclei in islets embedded in bioink were significantly increased after 4 days of culture, and this trend was preserved on the 7th day of culture. Interestingly, the insulin was detected only in the area of islets suggesting the limited function of the scaffolds. Wszola et al., in other studies, have shown that the functionality of pancreatic islets in bioprinted 3D structures is influenced by the composition of the bioink itself, as well as the origin of matrix proteins. The team conducted tests using an alginate-based bioink and the proprietary dECM (decellularized extracellular matrix) based bioink. Gelatin or commercially available extracellular matrix proteins (hyaluronic acid, collagen, laminin) were also added to the hydrogel (3% alginate). The results showed that no bioink composition was as effective as a hydrogel based on the dECM of the pancreas. Pancreatic islets, in the case of the produced bioink, were functional for 7 days. Such results have not been obtained for any other bioink. The GSIS test confirmed that for pancreatic islets, the effectiveness of the dECM-based bioink [19,49].

The authors have decided to double-check the islet condition and secretory function after the isolation procedure. For this purpose, we chose the kidney as the site of transplantation of isolated islets. The functional standpoint has provided us with the possibility of providing suitable metabolic conditions. Insulin secretion has been found in post-surgery kidneys, confirming the optimal isolation condition. The obtained results indicate that bioprinting of bionic scaffolds with biological material (pancreatic islets or cells of other tissues) may, in the future, become an alternative to traditional transplants. In this study, the authors compared the innovative bioprinting method with the well-known islet transplantation under the renal capsule. Thus, demonstrating another milestone for future transplantology. As can be seen, the reconstruction of the extracellular matrix made it possible to maintain the intended effect for a much longer time.

## 5. Conclusions

The presented results can be considered significant progress in the field of bioprinting of bionic structures with pancreatic islets.

The study showed that the transplantation of porcine pancreatic islets in the form of 3D bionic scaffolds into NOD-SCID mice without induced diabetes lowers the average glucose level and inhibits the functions of mice observed as lower values of mouse C-peptide in the study groups, similar to the group with islet transplantation under the renal capsule. In both groups, the positive effect of transplantation was maintained until the end of the experiment; however, in the 3D-bioprint group, the results were significantly lower compared to subcapsular transplantation.

To sum up, bioink A is a biocompatible material and allows for safe use in models of transplanted tissues. The proposed solution may, in the future, be used as a new therapy for people with type 1 diabetes or chronic pancreatitis. In addition to revolutionizing transplant medicine, bionic models may also become a new branch of pharmacological research in the future.

## 6. Patents

PCT/IB2020/056856—“Detergent-free decellularized extracellular matrix preparation method and bioinks for 3D printing”. 

## Figures and Tables

**Table 1:** Statistical analysis of glucose measurements in all study groups.

Control	Day 0	Day 7	Day 14	Day 28
**Day 0**	-	0.7527	0.8746	0.3526
**Day 7**	0.7527	-	0.6374	0.5313
**Day 14**	0.8746	0.6374	-	0.2813
**Day 28**	0.3526	0.5313	0.2813	-
**IsletTX**	**Day 0**	**Day 7**	**Day 14**	**Day 28**
**Day 0**	-	**0.0194**	0.0883	0.3316
**Day 7**	**0.0194**	-	0.4156	0.1175
**Day 14**	0.0883	0.4156	-	0.4156
**Day 28**	0.3316	0.1175	0.4156	-
**3D bioprint**	**Day 0**	**Day 7**	**Day 14**	**Day 28**
**Day 0**	-	**<0.0001**	**<0.0001**	**<0.0001**
**Day 7**	**<0.0001**	-	**<0.0001**	**<0.0001**
**Day 14**	**<0.0001**	**<0.0001**	-	0.5750
**Day 28**	**<0.0001**	**<0.0001**	0.5750	-

Statistically significant values are marked in red.

**Table 2:** Statistical analysis of C-peptide measurements in all groups.

Control	Day 0	Day 7	Day 14	Day 28
**Day 0**	-	0.1586	0.0576	0.0653
**Day 7**	0.1586	-	0.5619	0.6092
**Day 14**	0.0576	0.5619	-	0.9441
**Day 28**	0.0653	0.6092	0.9441	-
**IsletTX**	**Day 0**	**Day 7**	**Day 14**	**Day 28**
**Day 0**	-	**0.0001**	0.2605	0.1774
**Day 7**	**0.0001**	-	**0.0010**	**0.0016**
**Day 14**	0.2605	**0.0010**	-	0.8053
**Day 28**	0.1774	**0.0016**	0.8053	-
**3D bioprint**	**Day 0**	**Day 7**	**Day 14**	**Day 28**
**Day 0**	-	**<0.0001**	**0.0005**	**0.0002**
**Day 7**	**<0.0001**	-	0.3223	0.7562
**Day 14**	**0.0005**	0.3223	-	0.4885
**Day 28**	**0.0002**	0.7562	0.4885	-

Statistically significant values are marked in red.

## Data Availability

All data generated or analyzed during this study are included in this published article.
